# Measuring Social Motivation Using Signal Detection and Reward Responsiveness

**DOI:** 10.1371/journal.pone.0167024

**Published:** 2016-12-01

**Authors:** Coralie Chevallier, Natasha Tonge, Lou Safra, David Kahn, Gregor Kohls, Judith Miller, Robert T. Schultz

**Affiliations:** 1 Center for Autism Research, Children’s Hospital of Philadelphia, Philadelphia, PA, United States of America; 2 Laboratoire des Neurosciences Cognitives (LNC), INSERM U960, ENS-PSL, Paris, France; 3 Center for Cognitive Neuroscience, University of Pennsylvania, Philadelphia, PA, United States of America; 4 Child Neuropsychology Section, Department of Child and Adolescent Psychiatry, Psychosomatics and Psychotherapy, RWTH Aachen University, Aachen, Germany; 5 Departments of Pediatrics and Psychiatry, Perelman School of Medicine, University of Pennsylvania, Philadelphia, PA, United States of America; "INSERM", FRANCE

## Abstract

**Background:**

Recent trends in psychiatry have emphasized the need for a shift from categorical to dimensional approaches. Of critical importance to this transformation is the availability of tools to objectively quantify behaviors dimensionally. The present study focuses on social motivation, a dimension of behavior that is central to a range of psychiatric conditions but for which a particularly small number of assays currently exist.

**Methods:**

In Study 1 (N = 48), healthy adults completed a monetary reward task and a social reward task, followed by completion of the Chapman Physical and Social Anhedonia Scales. In Study 2 (N = 26), an independent sample was recruited to assess the robustness of Study 1’s findings.

**Results:**

The reward tasks were analyzed using signal detection theory to quantify how much reward cues bias participants’ responses. In both Study 1 and Study 2, social anhedonia scores were negatively correlated with change in response bias in the social reward task but not in the monetary reward task. A median split on social anhedonia scores confirmed that participants with high social anhedonia showed less change in response bias in the social reward task compared to participants with low social anhedonia.

**Conclusions:**

This study confirms that social anhedonia selectively affects how much an individual changes their behavior based on the presence of socially rewarding cues and establishes a tool to quantify social reward responsiveness dimensionally.

## Background

One of the great challenges in understanding the biological cause of psychiatric disorders has been that diagnostic categories based on clinical consensus do not align with findings from neuroscience and genetics [1]. In response to this challenge, it has become clear that it is necessary to go beyond traditional diagnostic boundaries by characterizing individual phenotypes dimensionally [2]. One dimension of behavior that is particularly well-suited to dimensional approaches is social motivation. Social motivation can be described as a set of biological mechanisms biasing the individual to preferentially orient to the social world and to treat social interactions as rewarding. Social motivation has been identified as a relevant behavioral dimension for a number of disorders including schizophrenia-spectrum disorders (e.g., [3]), anorexia nervosa (e.g., [4]), depression (e.g., [5]), psychopathy (e.g., [6]), and, perhaps most notably, autism spectrum disorders (ASDs) (e.g., [7]). However, the presence or absence of social motivation deficits does not directly align with any of these diagnostic categories, which suggests that social motivation is best construed in a dimensional framework cutting across multiple conditions.

Research investigating social motivation has been hindered by the paucity of tools to reliably quantify this construct. Most researchers aiming to quantify social motivation as a dimension indeed rely on a handful of self-reports instruments, such as the Chapman Social Anhedonia Scale [8] or the Affiliative Tendency Scale [9], which provide a useful first-pass evaluation of social motivation in typical adults but might be biased in both psychiatric and control populations. Individuals with a psychiatric condition may indeed lack self-reflection skills and struggle to accurately report on their own feelings [10] and control participants may be susceptible to social desirability effects leading them to overestimate their social motivation [11]. Another concern associated with existing self-report instruments is that they do not specifically target social reward responsiveness and instead combine various aspects of social motivation that might be best construed as distinct traits (e.g., social seeking, social attention, reputation management, gregarious instincts, social reward responsiveness, etc.).

In this paper, our goal is to use signal detection theory to create an objective tool tapping social reward responsiveness specifically. With this goal in mind, we adapt a task developed by Pizzagalli et al. to assess non-social reward responsiveness and its relationship to depression [12]. Specifically, previous work on non-social reward responsiveness has demonstrated that unequal frequency of reward is associated with a preference for the response that is associated with the more frequent reward [13,14] and that this response bias constitutes a reliable proxy of reward responsiveness [12]. Pizzagalli et al. (2005) then demonstrated that participants with elevated levels of depression symptoms showed a blunted response bias towards frequently rewarded stimuli, thereby demonstrating that their behavior was less modulated by reinforcement than individuals with low levels of depression symptoms. Here, we present two studies that extend this work to social reward responsiveness: Study 1 includes a monetary rewards task, which essentially replicates Pizzagalli’s task, and our social rewards task. We predicted that both a high frequency of social and monetary rewards would bias participants to answer faster and more accurately to the rich stimulus. More importantly, we predicted that individual differences in this response bias would correlate with self-reported social anhedonia but not with self-reported physical anhedonia, which refers to pleasures associated to non-social sensory experiences (e.g., eating, touching pleasant material, moving, experiencing smells and sounds, etc.). In Study 2, we ran a direct replication of the social task with an unrelated sample of healthy participants.

## Study 1: Designing a dimensional tool to assess social motivation

### Methods

#### Ethics Statement

This study was reviewed and approved by the Children’s Hospital of Philadelphia’s Institutional Review Board (IRB 11–008173). After complete description of the study to the subjects, written informed consent was obtained.

#### Materials

The monetary and social reward tasks were presented on a 22-inch widescreen monitor using E-prime. In the Money experiment, monetary rewards were presented as the plain text “+ 5 cents”, which represented a real 5-cent reward that participants were told would be added to their total reimbursement. In the social experiment, social rewards were in the form of a silent, full-color video clip of an actor providing approval by simultaneously smiling, nodding, and showing a thumbs-up gesture [15]. The rewards were provided in response to correct identification of a line appearing in the center of a circle as being short (11.5mm) or long (13mm).

Following the computer tasks, participants were left alone to complete the Beck Depression Inventory-II [16] and the Chapman Social and Physical Anhedonia Scales [8]. The BDI-II was used to screen out clinically depressed participants who might not have been identified during our phone screening. The Chapman scales were included in order to measure self-reported anhedonia in response to social and physical situations. The Social Anhedonia Scale measures how much participants find social interactions to be rewarding (e.g., the interpersonal pleasure of being with people, talking, exchanging expressions of feelings, doing things with others, competing, loving, and interacting in multiple other ways). Note that due to a computer error, only the first 32 items were administered in Study 1. 12 participants were re-invited to take the full version of the questionnaire within 4 months of their initial visit. Scores on the 32-item version and on the full 40-item version were highly correlated, *r* = .86, *p* = .0002. The Physical Anhedonia Scale measures how much participants find physical situations to be rewarding (e.g., physical pleasures linked to eating, touching, feeling, temperature, movement, smell and sound). All experimental scripts, analysis scripts, test materials and raw data are available for academic purposes on the Open Science Framework.

#### Participants

Forty-eight participants (27 female, 21 male, mean age: 24.16 +/- 3.28 years) were recruited from the Philadelphia area using Craigslist and paper flyers. Interested volunteers were screened over the phone to determine eligibility. Individuals who reported an active Axis I disorder or current psychotropic medication use were not invited to participate in the study. All participants received compensation for their time ($10 per hour) and travel costs, as well as a variable monetary reward depending on task performance ($5–6). Three participants with a task accuracy of 2 SD below the mean and an additional two participants above the clinical cutoff on the BDI-II were excluded from final analysis, leaving a final sample of 44 participants (19 males, 25 females).

#### Design and Procedure

Each participant completed both the Social experiment and the Money experiment, order counterbalanced across participants. Each experiment lasted approximately 25 minutes separated by a 10-minute categorization task that was unrelated to the current study. Participants were tested individually and stayed alone in the testing room during the entire duration of the experiment. The experimenter was only present to introduce each experiment and provide feedback during the training phase. The instructions were read aloud to the participants as they were viewing them simultaneously on the screen. Participants were told that their task would be to classify a line as either short or long by pressing the corresponding key and that feedback for correct responses would only occur some of the time. Depending on the experiment, participants were told that feedback for correct responses would be a “thumbs up” video clip (social reward task) or the text “+ 5 cents” (monetary reward task). The training phase consisted of five practice trials during which no reward and no feedback were provided.

**Fig 1:**
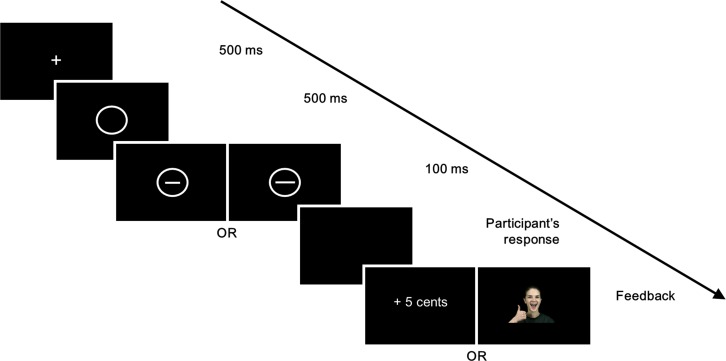
Schematic representation of the tasks. A fixation cross appears for 500ms, followed by an empty circle. A short or a long line is then flashed inside the circle for 100ms. Participants have an infinite amount of time to respond before they receive a reward for some of their correct responses.

Each experiment consisted of 300 trials separated into 100-trial blocks. Each trial began with the presentation of a fixation cross (500ms), followed by an empty circle (500ms). The short or the long line was then flashed within the circle (100ms) then disappeared to show a plain black screen during which participants could submit their response (see Fig 1). An equal number of short and long lines were presented within each block. Short and long lines were presented in a random order. Participants were given an infinite amount of time to indicate their response using ‘e’ or ‘p’ on the keyboard. Responses were followed by 1750ms of either a blank screen, or the social or monetary reward described above.

An asymmetrical reinforcement ratio was introduced such that one type of line (i.e. short or long) was rewarded more often. The line type that was rewarded more frequently is referred to as the “rich stimulus” and the line type that was rewarded least is referred to as the “lean stimulus”. Within each 100-trial block, participants received positive feedback on 30 trials for accurately identifying the rich line, and on 10 trials for accurately identifying the lean line. An algorithm was programmed so that reward feedback followed a pseudorandom schedule specifying which specific trials were to be rewarded for correct identifications [12,17]. If a participant failed to correctly identify the line on a trial programmed to get a reward, the reward was delayed until a line of the same type appeared and was correctly identified. The long line was randomly assigned to being the rich or the lean stimulus for each task and participant. Participants earned approximately $6 for the entire study.

#### Data Analyses

Our analysis plan was based on Pizzagalli et al. (2005) who relies on signal detection theory [18] to quantify reward responsiveness. In signal detection tasks, participants’ responses are classified into one of four categories: hits, false alarms, misses and correct rejections. In the experimental condition where the long line is the rich stimulus, hits refer to correct identifications of the long line as long, false alarms refer to identifications of the short line as long, misses refer to identification of the long line as short and correct rejections refer to identification of the short line as short.

Two conceptually distinct metrics can then be computed: 1) ‘discriminability’, which refers to participants’ ability to differentiate the two stimuli, and will be high if there are more hits and correct rejections than misses and false alarms; 2) ‘response bias’, which refers to the likelihood of giving one response more frequently than another and will be positive if there are more hits and false alarms than misses and correct rejections. High discriminability scores indicate increased ease of telling the two target stimuli apart and thus works as an index of task difficulty. Larger positive response bias scores indicate an increased tendency to identify the stimulus line as the more rewarded line and, simultaneously, a decreased tendency to identify the stimulus as the less rewarded line. Put another way, a large response bias indicates that an individual is more responsive to rewards.

Discriminability and response bias were calculated based on previous behavioral models of signal detection [12,19].

Discriminability was computed as:

$$Log\left(d\right)=1/2\;*\;log\left[\left(Rich_{correct}\;*\;Lean_{correct}\right)/\;\left(Rich_{incorrect}\;*\;Lean_{incorrect}\right)\right]$$

Response bias was computed as:

$$Log\left(b\right)=1/2\;*\;log\left[\left(Rich_{correct}\;*\;Lean_{incorrect}\right)/\;\left(Rich_{incorrect}\;*\;Lean_{correct}\right)\right]$$

Rich _correct_ and Lean_correct_ correspond to the proportion of correct identifications (hits and correct rejections) to the total number of rich and lean trials respectively, and Rich_incorrect_ and Lean _incorrect_ correspond to the proportion of false identifications (misses and incorrect rejections) to the total number of rich and lean trials respectively. When accuracy was equal to 1 or 0, we followed the log linear correction procedure described by Hautus et al. [20].

Accuracy, RT and discriminability were analyzed in order to assess overall performance for each experiment separately (Money and Social) using two-way ANOVAs on accuracy, RT and discriminability scores with Condition (Rich, Lean), and Block (1, 2, 3) as within-subjects factors. No main effect or interaction involving gender was found; we therefore present results for both genders combined in all our analyses. As in Pizzagalli et al. (2005), trials with a reaction time shorter than 150 ms, longer than 2500 ms, or ± 3 SDs from the mean (after natural logarithm transformation) were excluded from all analyses (3.6% of the trials overall).

Our main variable of interest was response bias because it reflects participants’ responsiveness to monetary rewards or social rewards. Following Pizzagalli et al. (2005), overall change in response bias (or ΔResponse Bias) was conceptualized as the difference between the first block and the last block. We first ran a repeated-measures ANOVA with Block as a within-subject factor to test whether Response Bias changed over time. We then examined the relationship between anhedonia and ΔResponse Bias to either social or monetary rewards using correlations, a median-split based on participants’ social anhedonia scores.

### Results

#### Task 1: Social rewards task

**Fig 2:**
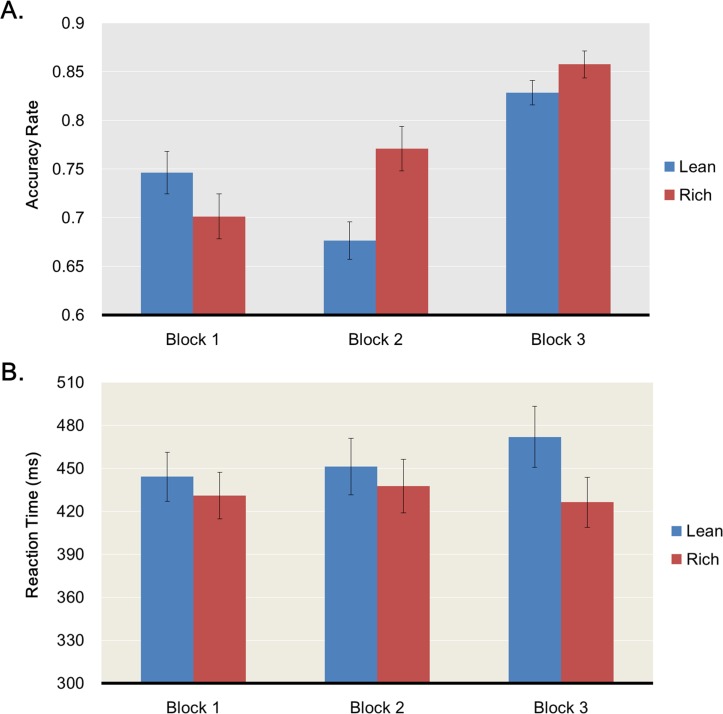
Performance in the social reward task. Accuracy rates (A) and reaction times (B) during the social reward task. Results are split by block (1, 2, 3) and stimulus type (lean in blue, rich in red).

Overall task performance (Fig 2). A two-way ANOVA on accuracy scores with Condition (Rich, Lean), and Block (1, 2, 3) as within-subjects factors revealed a main effect of Condition, *F*(1,43) = 5.55, *p* < .023, η_p_^2^ = .114, a main effect of Block, *F*(2,42) = 30.01, *p* < .001, η_p_^2^ = .588, and a Condition x Block interaction, *F*(2,42) = 9.616, *p* < .001, η_p_^2^ = .314. As predicted, accuracy for the rich condition (*M* = .777, *SD* = .090) was higher than for the lean condition (*M* = .750, *SD* = .082), *t*(43) = 2.355, *p* = .023, *d* = .32, and increased over time (Block 1 vs. 2, *t*(43) = 0.002, *p* = .998; Block 2 vs. 3, *t*(43) = -7.83, *p* < .001; Block 1 vs. 3, *t*(43) = -4.91, *p* < .001). The ANOVA on reaction times revealed a main effect of Condition, *F*(1,43) = 23.45, *p* < .001, η_p_^2^ = .353, no main effect of Block, *F*(2,42) = 0.244, *p* = .785, η_p_^2^ = .011, and a Condition x Block interaction, *F*(2,42) = 5.417, *p* = .008, η_p_^2^ = .205. Reaction times for the rich condition (*M* = 431.7ms, *SD* = 103.2ms) were faster than in the lean condition (*M* = 455.8ms, *SD* = 116.0ms), *t*(43) = -4.84, *p* < .001. Planned comparisons revealed that the Condition x Block interaction was due to participants being faster in Block 3 for the rich condition, *t*(43) = 5.78, *p* < .001, but not in the other two blocks, Block 1: *t*(43) = 1.84, *p* = .073, Block 2: *t*(43) = 1.67, *p* = .103. Finally, the ANOVA on discriminability with Block as a within-subject factor revealed no main effect, *F*(2,42) = 1.203, *p* = .305, η_p_^2^ = .027.

**Fig 3:**
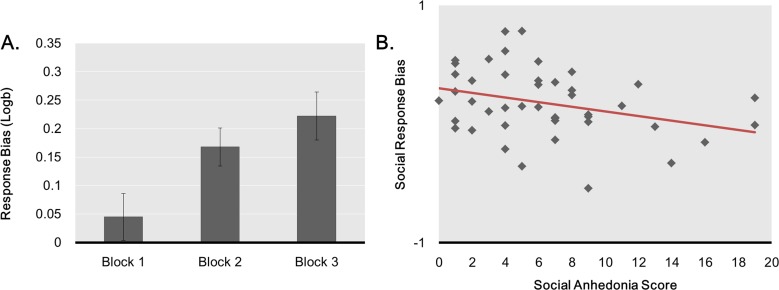
Response bias in the social reward task. Evolution of the response bias across blocks in the social reward task (A) and correlation between change in response bias (i.e. Logb Block 3 –Logb Block 1) and social anhedonia score, *R*^*2*^ = .116.

**Response bias** (Fig 3). In line with our hypothesis that response bias would increase over time, a repeated-measures ANOVA on response bias with Block as a within-subject factor revealed a main effect of Block, *F*(2,42) = 7.77, *p* = .001, η_p_^2^ = .270, due to an increase in response bias from Block 1 to Block 2, *t*(43) = -3.081, *p* = .004, and from Block 1 to Block 3, *t*(43) = -3.902, *p* < .001. Pearson correlations revealed that ΔResponse Bias did not correlate with physical anhedonia scores, *r* = -.044, *p* = .774, *n* = 44 but correlated with social anhedonia scores, *r* = -.340, *p* = .024, n = 44, indicating that participants with stronger self-reported social anhedonia were also the least biased by social rewards. By contrast, social anhedonia scores and physical anhedonia scores strongly correlated, *r* = .442, *p* = .003, *n* = 44, suggesting that the specificity of each instrument should be questioned and that ΔResponse Bias might be a more accurate measure of social anhedonia than a commonly used self-report measure.

Using a standard categorical approach, we then compared change in response bias in higher and lower social anhedonia participants. Specifically, we looked at the difference in response bias between Block 1 and 2 as well as between Block 1 and 3. Change in response bias was greater for lower social anhedonia participants than for higher social anhedonia participants for the Block 1 vs. Block 2 comparison, *t*(42) = 2.409, *p* = .020, and for the Block 1 vs. Block 3 comparison, *t*(42) = 2.627, *p* = .012. Importantly, Participants in the lower social anhedonia group (score range: 0–6, *M* = 3.24, *SD* = 1.92, N = 25) and a higher (score range: 7–19, *M* = 10.47, *SD* = 3.98, N = 19) and in the higher social anhedonia group did not differ with respect to age, *t*(42) = 0.426, *p* = .672, Gender ratio, χ^2^(1) = 0.548, *p* = .547, percentage of outliers, *Z* = -0.996, *p* = .319, Mann-Whitney U, or number of feedbacks received during the experiment, Money: *t*(42) = 0.206, *p* = .838; Social: *z* = -.326, *p* = .744, Mann-Whitney U. Finally, the two groups did not differ in the rich-to-lean reward ratio, which is the most important variable that drives response bias (Money task: *t*(42) = 0.709, *p* = .483; Social Task: *t*(42) = 1.239, *p* = .222).

#### Task 2: Monetary rewards responsiveness

**Fig 4:**
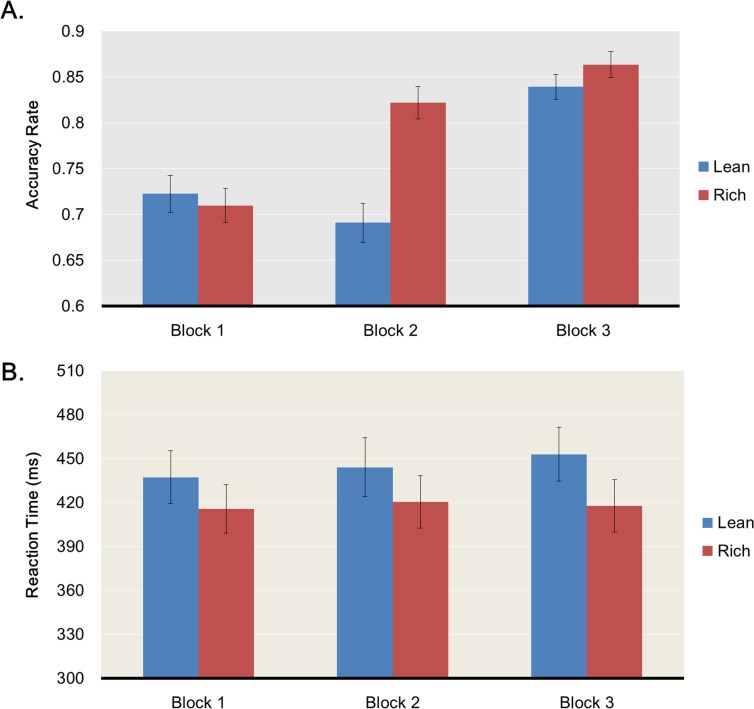
Performance in the monetary reward task. Accuracy rates (A) and reaction times (B) during the monetary reward task. Results are split by block (1, 2, 3) and stimulus type (lean in blue, rich in red).

**Overall task performance** (Fig 4). A two-way ANOVA on accuracy scores with Condition (Rich, Lean) and Block (1, 2, 3) as within-subjects factors revealed a main effect of Condition, *F*(1,43) = 16.15, *p* < .001, η_p_^2^ = .27, a main effect of Block, *F*(2,42) = 39.17, *p* < .001, η_p_^2^ = .65, and a Condition x Block interaction, *F*(2,42) = 9.37, *p* < .001, η_p_^2^ = .308. As predicted, accuracy for the rich condition (*M* = .798, *SD* = .067) was higher than for the lean condition (*M* = .751, *SD* = .095), *t*(43) = 4.019, *p* < .001, and increased over time (Block 1 vs. 2, *t*(43) = -2.277, *p* = .028; Block 2 vs. 3, *t*(43) = -6.72, *p* < .001; Block 1 vs. 3, *t*(43) = -7.97, *p* < .001). The ANOVA on reaction times revealed a main effect of Condition, *F*(1,43) = 25.95, *p* < .001, η_p_^2^ = .376, no main effect of Block, *F*(2,42) = 0.405, *p* = .670, η_p_^2^ = .019, and no Condition x Block interaction, F(2,42) = .842, *p* = .438, η_p_^2^ = 0.039. Reaction times for the rich condition (*M* = 417.9ms, *SD* = 105.9ms) were faster than for the lean condition (*M* = 444.9ms, *SD* = 118.1ms), *t*(43) = -5.09, p < .001. Finally, the ANOVA on discriminability with Block as a within-subject factor revealed no main effect, *F*(2,42) = 0.606, *p* = .548, η_p_^2^ = .014.

**Fig 5:**
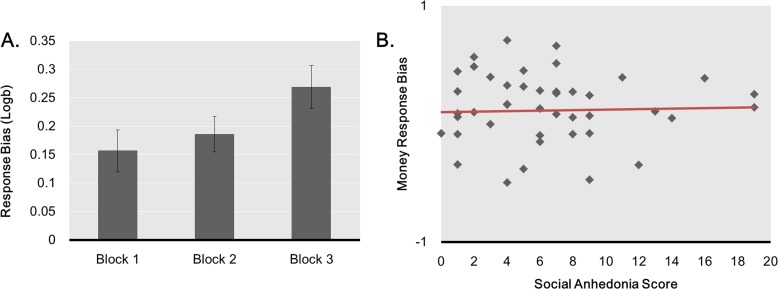
Response bias in the monetary reward task. Evolution of the response bias across blocks in the monetary reward task (A) and correlation between change in response bias (i.e., Logb Block 3 –Logb Block 1) and social anhedonia score, *R*^*2*^ = .058.

**Response bias** (Fig 5). In line with our hypothesis that response bias would increase over time, a repeated-measures ANOVA on response bias with Block (1, 2, 3) as a within-subject factor revealed a trend for a main effect of Block, *F*(2,42) = 2.955, *p* = .057, η_p_^2^ = .064, due to an increase in response bias from Block 1 to Block 3, *t*(43) = -2.105, *p* = .041 and from Block 1 to Block 2, *t*(43) = -7.594, *p* < .001. Unlike what we found in Task 1, ΔResponse Bias correlated neither with social anhedonia scores, *r* = .024, *p* = .875, *n* = 44, nor with physical anhedonia scores *r* = .030, *p* = .849, n = 44. Similarly, we found no difference between higher and lower social anhedonia participants on ΔResponse Bias for Block 1 vs. Block 2, *t*(42) = -0.148, *p* = .883, or for Block 1 vs. Block 3, *t*(42) = -0.432, *p* = .668. We had no hypothesis regarding the correlation of ΔResponse Bias between the Money and Social task but our exploratory analysis revealed a weak correlation, *r* = .28, *p* = .066.

## Study 2: Direct Replication Study

In order to ensure the robustness of the relationship between social anhedonia and ΔResponse Bias we ran the same study in an independent sample of participants.

### Methods

#### Ethics Statement

This study was reviewed and approved by the local ethics committee (Comité de protection des personnes Ile de France III). After complete description of the study to the subjects, written informed consent was obtained.

#### Participants

26 French participants (14 females and 12 males) completed the Social experiment described in Study 1. As in Study 1, participants were left alone after the computer task to complete the French version of the Beck Depression Inventory [21] and the Chapman Anhedonia Scales [22] Participants earned approximately 10€ for the entire task.

#### Analyses

No difference between males and females was found in any of the variables of interest, we therefore present results for both genders combined in all our analyses. As in Study 1, trials with a reaction time shorter than 150 msec, longer than 2500 msec or ± 3 SDs from the mean (after natural logarithm transformation) were excluded from all analyses (2.1% of the trials overall). Discriminability and response bias were calculated using the same formulae as in Study 1.

### Results

#### Overall task performance

A two-way ANOVA on accuracy scores with Condition (Rich, Lean), and Block (1, 2, 3), as within-subjects factors revealed a main effect of Condition, *F*(1,25) = 11.29, *p* < .01, η_p_^2^ = .31, a main effect of Block, *F*(2,24) = 8.11, *p* < .01, η_p_^2^ = .40, but no Condition x Block interaction, *F*(2,24) = 1.41, *p* = .26, η_p_^2^ = .10. As predicted, accuracy for the rich condition (*M* = .853, *SD* = .101) was higher than for the lean condition (*M* = .760, *SD* = .153), *t*(25) = 3.36, *p* < .01. The ANOVA on reaction times revealed a main effect of Condition, *F*(1,25) = 13.20, *p* < .01, η_p_^2^ = .346, no main effect of Block, *F*(2,24) = 0.42, *p* = .661, η_p_^2^ = .034, and no Condition x Block interaction, *F*(2,24) = 1.47, *p* = .249, η_p_^2^ = 0.109. Reaction times for the rich condition (*M* = 466.9ms, *SD* = 124.5ms) were faster than for the lean condition (*M* = 491.1ms, *SD* = 134.6ms), *t*(25) = -3.63, *p* < .01. Finally, the ANOVA on discriminability with Block as a within-subject factor revealed a main effect of Block, *F*(2,24) = 11.024, *p* < .001, η_p_^2^ =. 408.

#### Response bias

A repeated-measures ANOVA on response bias with Block (1, 2, 3) as a within-subject factor revealed no main effect of Block, *F*(2,24) = 0.808, *p* = .452, η_p_^2^ = .119. Even though response bias is computed to be independent from discriminability, we checked for the presence of any significant correlation between these two variables. These analyses revealed no significant correlation between discriminability and response bias: *r* = -.194, *p* = .343, n = 26, as well as no significant correlation between discriminability and ΔResponse Bias: *r* = .092, *p* = .653, n = 26. As found in Experiment 1 of Study 1, ΔResponse Bias did not correlate with physical anhedonia scores *r* = -.105, *p* = .609, n = 26 but correlated with social anhedonia scores, *r* = -.462, *p* = .017, *n* = 26.

We then compared change in response bias in higher and lower social anhedonia participants by creating a median-split based on participants’ scores in the Chapman social anhedonia scale. We separated our sample into a lower (range: 2–7, n = 13) and a higher (range: 8–27, n = 13) social anhedonia Group. Lower social anhedonia (*M* = 5.46, *SD* = 1.85) and Higher social anhedonia (*M* = 14.15, *SD* = 5.67) subjects did not differ with respect to Gender ratio, *χ^2^*(1) = 0.002, *p* = .962, percentage of outliers, *Z* = -.515, *p* = .621, Mann-Whitney U, number of feedbacks received during the experiment: *Z* =. 506, *p* = .673, Mann-Whitney U, or rich-to-lean reward ratio, *t*(24) = 1.239, *p* = .222. The two groups differed on age, with the Higher social anhedonia group being older than Lower social anhedonia, *t*(24) = 2.24, *p* = .034. However, age did not correlate with ΔResponse Bias, *r* = -.303, *p* = .132, n = 26, and ΔResponse Bias still correlated with Social Anhedonia Scores when controlling for Age, *r* = -.395, *p* = .039, n = 26.

We then looked at the difference in response bias between Block 1 and 2 as well as between Block 1 and 3. Change in response bias was not significantly greater for lower social anhedonia participants than for higher social anhedonia participants for the Block 1 vs. Block 2 comparison, *t*(24) = 0.505, *p* = .618, but was significantly greater for lower social anhedonia participants than for higher social anhedonia participants for the Block 1 vs. Block 3 comparison, *t*(24) = 2.180, *p* = .039.

## Discussion

Lack of reliable and objective measures available to assess social motivation is likely to be an important barrier in understanding the biological and genetic roots of a number of psychiatric disorders in which disrupted social motivation is commonly reported [7]. In this study, social rewards presented in the context of a signal detection task were found to positively bias performance in two independent healthy adult samples, which replicates and extends prior findings using monetary rewards [12]. Most importantly, individual differences in the strength of this social bias correlated with self-reported social motivation as measured with the social anhedonia scale. In other words, the behavior of participants who reported taking less pleasure in social interactions was less affected by social rewards.

Dimensional approaches to psychiatry have recently been encouraged as a necessary paradigm shift in order to further our understanding of the biological bases of a range of disorders. Indeed, genetic approaches rely on careful characterization of aberrant phenotypes to infer the consequence of genetic mutations; and in a similar way, neuroscientists infer the function of specific brain areas by relating brain activity to the subject’s behavior. The idea of breaking down clinical symptomatology into biologically relevant units of quantifiable behaviors also aligns with NIMH’s recent Research Domain Criteria (RDoC) initiative encouraging research on constructs that cut across diagnostic boundaries. As part of this initiative, working groups have been identifying promising and reliable research paradigms to study three key constructs in the social domain: i) affiliation and attachment (which encompasses social motivation), ii) social communication, iii) perception and understanding of self and others [23]. Interestingly, only three experimental paradigms are listed in the summary of the working groups as potential assays for affiliation and attachment, in comparison to more than 30 tools listed for each of the other two constructs. This highlights the great need to create finely graded dimensional tools for making predictions about genetic, neurobiological and other behavioral dimensions. It is clear that more measures are needed to fully develop a nomological network [24] of social reward and to validate concepts such as social motivation, affiliation and attachment.

One might however question the potential benefit of using behavioral measures rather than psychometric scales. In the context of our task, we found that the Social Anhedonia Scale is correlated with the Physical Anhedonia Scale whereas social reward responsiveness correlates specifically with social anhedonia (but not with physical anhedonia). This suggests that our task taps into a narrower—and hopefully better defined—construct than the one assessed by the Social Anhedonia Scale. Second, as we explained in the introduction, psychometric measures are limited in a number of ways: some people may lack insight and fail to accurately report their own social preferences and social skills, other individuals may be well-aware of their social limitations but may choose to under-report their issues to provide more socially desirable responses [11]. This concern applies to many items in the Chapman scale where there is an answer that is viewed more favorably by society (e.g., answering ‘No’ to: “I attach very little importance to having close friends”, “Playing with kids is a real chore”, “I don't really feel very close to my friends”). Given that the social desirability bias is not equally strong in all populations, this feature of psychometric scales assessing social motivation may amplify or distort group differences when studying participants who are less concerned with their reputation (e.g., [7]). or with societal norms (e.g., [6]).

Our behavioral task also differs from a number of existing paradigms, notably eye-tracking experiments in which it is difficult to tease apart the influence of participants’ preference for social stimuli from lower-level factors such as stimulus features [25] or individual differences in the visual system [26]. In designing our study, our goal was to make sure that participants’ behavior was truly guided by internal value signals rather than by low level features of the task. Similarly, a recent study by Dubey et al. on social seeking demonstrated that typical adults put in more conscious effort to view social vs. non social stimuli, which can be used as a proxy to measure social motivation [27]. We believe that our protocol, which focuses more specifically on social reward responsiveness rather than on social seeking, nicely complements this instrument, with the added value that participants have to make no conscious decision to react to the social signal. The implicit nature of the task indeed prevents social desirability biases from confounding the results.

There are, however, a number of limitations to the present study. First, it will be important to use this tool in clinical populations to assess its sensitivity to variations in social motivation in various psychopathologies; e.g. Is social reward responsiveness lower in Autism Spectrum Disorders, higher in Williams Syndrome, lower in depression, etc.? Another interesting empirical question will be to determine whether social reward responsiveness varies with participants’ social cognition abilities. It is indeed possible that individuals who are confused by social interactions do not respond to social rewards in the same way as individuals who are more socially gifted.

Ultimately, advancing the validation of adequate psychological tools that can reliably quantify subtle individual differences in social functioning is an important challenge to advance our knowledge of the genetics and neurobiology of psychiatric conditions. Behavioral tools measuring social motivation might also prove necessary for identifying subtypes for various disorders affected by social motivation impairments, including schizophrenia, ASDs, anorexia nervosa and depression and in gaining a better understanding of other clinically complex conditions. Reliable and finely graded dimensional measures will also make more sensitive predictions about clinically relevant outcomes, such as predicting risk and disorder onset, natural history, and differential response to various treatments. Combining diagnostic categories with dimensional description is therefore likely to be a fruitful paradigm, as it has been in most areas of medicine for many years [28]. As Rutter recently pointed out, the use of dimensional approaches will indeed allow us “to portray the true picture of a clinical presentation that involves multiple facets or straddles the boundary between two adjacent categories, without ending up with spurious comorbidity and without the necessity of forcing symptom patterns into a predetermined stereotype.” ([28], p. 655).
